# “It’s like building a new person”: lived experience perspectives on eating disorder recovery processes

**DOI:** 10.1186/s40337-024-01045-5

**Published:** 2024-07-08

**Authors:** Andrea LaMarre, Megan Hellner, Scout Silverstein, Jessica H. Baker, Bek Urban, Jacqlyn Yourell, Hannah Wolfe, Taylor Perry, Dori Steinberg

**Affiliations:** 1 Independent Researcher, Halifax, Canada; 2 Equip Health, 2659 State Street Suite 100 #1012, Carlsbad, CA 92008 USA

## Abstract

**Background:**

Deeply engaging with the expertise of those who have experienced or supported someone with an eating disorder can add to a growing body of knowledge about recovery processes. In this qualitative study, we sought to explore and generate nuanced understandings of recovery experiences of people with a lived ED experience (first hand or as a caregiver) who were working as mentors in the field. To do this, we focused on changes that occur in personality, traits, and interests over the course of an eating disorder and into recovery.

**Method:**

We conducted semi-structured interviews with 27 people with an eating disorder history, either through personal lived experience (n = 14) or as a caregiver of a loved one with an eating disorder (n = 13). We undertook a reflexive thematic analysis of the data through a critical realist lens.

**Results:**

We developed three themes, which illustrate the nonlinearity, relationality, and systemically linked nature of changes across experiences of having and recovering from an eating disorder. The first theme focuses on expansion; participants described how their worlds got bigger as they explored who they were becoming and discovered new ways of living in line with their values. The second theme emphasizes the balance between support and autonomy participants described as important for enabling change to occur across the recovery process. The last theme highlights the ways in which changes throughout the recovery process entwined with systemic factors, including actively pushing back against diet culture and weight stigma.

**Conclusions:**

Participants’ stories highlight interactions between individual, relational, and societal shifts that occur throughout the course of an ED and into recovery. They support ongoing calls to orient to ED recovery as situated within a broader social milieu, which invites us to build supportive environments to enable expansion and flourishing.

**Supplementary Information:**

The online version contains supplementary material available at 10.1186/s40337-024-01045-5.

## Abstract (plain language summary)

If we wish to better understand eating disorder recovery, it is important to ask those who have lived experience. In this qualitative study, we sought to better understand changes that people experience while they have an eating disorder and into recovery. We conducted interviews with 27 people with an eating disorder history. Participants had either first-hand lived experience or lived experience of supporting someone with an eating disorder, and were working as peer (n = 14) or family (n = 13) mentors supporting others through recovery. We analyzed this data using reflexive thematic analysis. We developed three themes, which together paint a picture of recovery as a non-linear experience that occurs with the support of others. First, participants shared how recovery felt like the world was getting bigger as they discovered a version of themselves that resonated with their values. Second, they discussed the importance of striking a balance between being supported and having autonomy. Finally, participants reflected on how they needed to push back against weight stigma and diet culture as they worked toward recovery. These stories help to illustrate the importance of looking at shifts and changes in recovery to better understand it—including individual shifts, shifts in relationships, and social shifts that can support recovery.

**Supplementary Information:**

The online version contains supplementary material available at 10.1186/s40337-024-01045-5.

## Introduction

People with lived experience of an eating disorder (ED), either through personal experience or supporting someone with an ED, have unique perspectives on what “recovery” feels and looks like. Those who go on to mentor and support others who are struggling with an ED use their experiences in this work regularly; in so doing, they may reflect on the “key ingredients” that impacted their recovery journeys. Increasingly, the value of integrating lived experience perspectives into the treatment and support continuum is being recognized [1, 2]. Lived experience support has been illustrated to be particularly helpful for increasing hope for recovery and improving quality of life [3, 4]. Studies about the value of lived experience in treatment and support contexts include an array of modalities (e.g., peer support, sharing recovery narratives, etc.) [1] and often include the perspectives of both those giving and receiving lived experience support [3]. There is room to further explore how those providing lived experience support are conceptualizing and exploring their own recovery journeys.

Lack of definitional clarity around what constitutes ED recovery persists in the field [5, 6]. Efforts to delineate a consensus definition of recovery have been underway for decades [7, 8]. However, stakeholders (e.g., people in recovery, clinicians, supporters, etc.) sometimes differ in their orientation to recovery [9]. Explorations of recovery that integrate lived experience perspectives have emphasized the importance of considering quality of life [10] and explored how while symptom remission may be a critical piece of the puzzle, subjective experiences of recovery do not necessarily begin and end there [11].

ED recovery is often understood as a process [12, 13] and as an identity journey [14]. Thinking about recovery in this way enables a perspective that positions a self in recovery that is not fully “finished”—and that is built from *both* treatment and societal experiences. People recovering from an ED do so in relation to others in their lives and social contexts more broadly [15]; supportive relationships are often described as core to recovery experiences [16]. They often recount their experiences of navigating environments saturated with diet culture [17–19] and weight stigma [20]. As people recover from EDs and reflect on their experiences, *how* they talk about recovery is partly informed by their social positioning, treatment modalities they have encountered, and broader social narratives about what recovery is [21, 22]. Some recovery stories are given more space than others; this can lead to particular, often privileged (e.g., white, able-bodied, cisgender, thin, etc.) versions of recovery coming to represent “the” story of recovery [23–25]. To understand what recovery looks like, it is helpful to understand more about the particular person seeking recovery—to gain insight into the context of their lives and relationships [15, 26].

When looking at the changes that occur over the course of an ED and into recovery, it is helpful to consider how the recovering self is conceptualized. Bardone-Cone and colleagues describe how “eating disorders have been intimately connected with the self and identity” (p. 60) [27]. Self-concept and its interrelated constructs (e.g., self-esteem, self-efficacy, selflessness, self-oriented perfectionism, self-criticism, etc.) have been implicated in the etiology and course of EDs and in relation to comorbidities [27]. In recovery, aspects of self—including constructs such as personality, and traits such as perfectionism and self-esteem—are considered to change, sometimes reaching levels close to or indistinguishable from those defined as healthy controls [28]. Aspects of self are also seen as factors that potentially impact the course and outcome of EDs [29]. Central to these analyses is the idea of a self as something that is relatively stable but modifiable with intervention. Approaches like these provide possible targets for intervention and treatment (for instance, temperament-based approaches) [30]. However, these approaches may not be able to explore in detail the less tangible aspects of self—the felt sense of who one “is.”

Taking qualitative approaches to studying the self in recovery allows for additional possibilities of envisioning how people construct themselves in relation to their social worlds. Several qualitative studies on recovery from EDs have suggested that the separation of illness and self *define* the recovery experience, taking a perspective that positions the “during an ED” and “after an ED” self as largely distinct [31, 32]. Williams and colleagues undertook a grounded theory study exploring how people with anorexia nervosa (AN) conceptualize self and how AN impacts their identity [33]. The authors suggested that AN “took over” the self during ED onset, after which point it took on a protective character and became shared with the person, and, eventually, something the person could not imagine themselves without. Recovery, then, involved a process of accepting fear of what the self might be without the AN [33]. This perspective foregrounds an orientation to self that positions the ED as a largely separate entity—if at times integrated into the person—that must be overcome in order to recover. However, this orientation risks *subsuming* the person with an ED into their illness in a way that makes recovery difficult to imagine [34]. Conti has also focused on the self in recovery, conducting a narrative analysis of the experiences of people in recovery from AN. Participants often framed recovery itself as a contested term with conflicting meanings as they reclaimed their identities through and following an ED [14]. Seen in this way, rediscovering self in recovery is not a straightforward task; it also involves re-thinking what recovery is and making meaning from experiences. Both Conti [14] and Malson [34] invite thinking about the identities that people moved *toward*—not as a return to pre-ED self, but as a self “acting differently in their lives,” (p. 84) [14] for instance through greater alignment with values.

In our study, we sought to better understand changes in personality, interests, and traits that occur across the course of an ED and into recovery. We did so by exploring the experiences of those with lived experience, either through personal experience or supporting a loved one through recovery. We specifically interviewed individuals known as mentors providing support to patients and families receiving family-based therapy (FBT) for an ED. Because mentors use their lived experiences in their work, they are likely to have extensively reflected on their recovery experiences—including through training and supervision [3]. We conceptualized self, personality, and identity as entangled with social worlds and relationships—and sought to understand these in relation to the different perspectives our participants foregrounded. While we draw on the idea of self in recovery, we focused on the process of recovery—the shifts and changes that arose as participants navigated the world while experiencing an ED and into recovery. We aimed to contribute to deepening understandings of ED recovery and informing recovery-focused interventions.

## Method

### Recruitment

We received IRB ethics approval through BRANY (Biomedical Research Alliance of New York). We recruited peer and family mentors currently employed at a virtual eating disorder treatment center. Mentors were assured that participation was voluntary and would not impact their employment. All mentors were eligible for participation, and there were no exclusions otherwise. For recruitment, a flier was shared electronically inviting mentors to participate in a study about the experience of recovering from an eating disorder. It was made clear that participation was voluntary and participants were provided with an honorarium in the form of a $25 Amazon gift card. Interested participants were instructed to scan a QR code where they were able to review and acknowledge an informed consent form electronically. Consenting participants were then contacted by research personnel to schedule an interview. Interviewees were employees of the same organization as some members of the research team; however, interviewees were members of the clinical team, whereas interviewers were members of the research team. We explained both in recruitment and prior to interviews that participation was voluntary and assured participants that quotes shared would be de-identified to the level that others working within the organization, as well as the general public, would not be able to identify them. Further, the lead author was an external, paid consultant.

### Participants

Participants were peer and family mentors. The peer mentor role involves regular meetings with the patient, offering support, using self‐disclosure as appropriate, and acting as a recovery role model. The family mentor offers support and strategies to the patient's caregivers around containment of ED behaviors and managing home-based refeeding efforts. Twenty-nine mentors responded to outreach; 27 were interviewed. Of these, 14 were peer mentors and 13 were family mentors. Participants predominantly identified as White, cisgender women; some participants identified as non-binary or as men. In order to preserve confidentiality due to the specificity of the sample we have used “they” as a pronoun for all participants in the results section.

In line with our methodological approach, we considered information power [35] to determine whether data were adequately rich. Our participants held a great deal of information about our research question, being experts over their own lived experiences. Analytically, we were interested in developing patterns of shared meaning across the dataset [36]. As we will describe, we were able to conduct separate thematic analyses on the basis of the detailed stories shared by each group of participants. Combining the analyses was a decision made in the next stage of analysis, based on the similarities between the analyses we conducted. As such, we were able to retain the richness of our in-depth analyses of participants with the most closely aligned experiences (e.g., peer mentors and family mentors) while then exploring patterns across both of these groups.

### Training and fidelity

All research team members undertook semi-structured interview training with AL. In this training, we worked together to finalize the interview guide and engaged in practice interviews. Seven members of the research team (SS, BU, TW, JY, HW, MH, and JB) conducted semi-structured interviews with participants; interviews took approximately 1 h.

### Procedures

The interview guide (Supplementary Materials) followed a similar structure for peer and family mentors, with some differences in phrasing. The initial interview guide was developed by MH and SS; over the course of several meetings, a group of subject matter experts, including individuals with lived experience, revised the guide. The practice interviews described above also led to additional adjustments in the guide prior to research interviews commencing. Interviews began by establishing shared terminology for EDs and recovery, which was used throughout the interview. Participants were then taken through a set of questions designed to explore personality, traits, and interests at different phases of their journeys, including recovery. Interview questions also explored whether participants felt like they ever returned to an “authentic” or “old” self, their relationships with food, weight, and physical activity throughout their experiences, what they felt contributed to the eating disorder and recovery, and where they were at in recovery.

Interviewers engaged in reflexive practice throughout the interview process. Each interviewer wrote a post-interview memo. Given the large size of the interviewing team, these memos provided helpful context throughout the analytic process. As a research team, we also considered the role that our different positionalities brought to the work of conducting interviews, engaging in analysis, and writing up findings. Authors AL, JY and HW were external research consultants. All other authors were affiliated with the organization through which the mentors were employed. Having a lead researcher external to the organization provided an outside perspective of internal organizational knowledge, while having researchers involved with the organization but from the research team offered inside perspectives on the organizational context. The research team is predominantly white and educated at varied higher education levels, from Bachelors to doctoral degrees. Seven authors are cisgender women and two authors are non-binary. Most have lived experience with eating disorders. 

### Data analysis

Interviews were transcribed using Zoom’s transcription feature and then checked manually against audio files of the interviews to correct any errors introduced in the automatic transcription process. We then undertook a reflexive thematic analysis (RTA) [36–38] of the interview data. Our analysis was informed by a critical realist framework, presuming that while there is a certain “reality” to experiences, we access mediated and contextualized interpretations of realities [39]. Initially, we conducted a separate thematic analysis of each set of interviews (peer mentors and family mentors). AL led this process, beginning with familiarization—close, repeated reading of each interview and note-taking about key points and impressions of the data. Next, she coded each transcript using MAXQDA software and generating codes as she progressed through coding. A separate coding process—and code list—was generated for the peer mentor set and the family mentor set. MH also coded the family mentor set using MAXQDA, and AL and MH met to discuss the coding process after coding several interviews. The purpose of this meeting was to enrich the coding process, rather than to generate precise agreement. Codes were refined throughout the process to minimize overlap while retaining nuance.

Next, AL began to develop themes in relation to the research questions, exploring patterns of shared meaning in the coded data. AL and MH met to discuss the developing themes; throughout this discussion and in reviewing the candidate themes for each set of interviews, AL and MH determined that there was significant overlap between the themes they had developed for the two groups. At this point, the decision was made to combine themes between the two groups to be able to tell a richer story inflected with nuances related to peer and family mentor participants’ experiences. Once AL and MH had generated a combined structure with 3 themes, these were shared back with the larger research team for their input and insights, as well as to explore whether and how the analysis resonated with their impressions from conducting the interviews. This step helped to establish the trustworthiness of the data by ensuring that themes resonated with those who had conducted the interviews, many of whom had lived experience of EDs as well. We also shared a summary of findings with participants. Analysis continued throughout the writing process as analytic insights were developed in bringing this article together.

#### A note about terminology

Not all participants identified with the term eating disorder, and not all used the word recovery to describe their experiences. ED diagnosis was not a requirement for inclusion; this was important to the research team particularly given the barriers to diagnosis and treatment for EDs, which disproportionately impact BIPOC and gender non-conforming and trans individuals [40–42]. When describing specific experiences in relation to quotes, we will use the individual’s preferred terminology. When writing about the results as a collective, we will use the terms ED and recovery.

## Results

We developed three themes from the data; these themes are distinct but work together to generate a picture of the process of changing and shifting throughout recovery experiences and the “ingredients” associated with this process of change. The first theme describes participants’ reflections on their or their loved one’s *expanding worlds* in recovery. Peer mentors in particular articulated experiences of becoming someone new; family mentors tended to see more of their loved one’s “old” self, but described this self as fundamentally changed by the ED. The second theme reflects a *balance of autonomy and support in the recovery process*—the importance of finding ways to support and be supported into recovery, throughout the shifts and changes participants experienced. Given the ways in which the ED—and, at times, treatment experiences—interrupted trust, finding this balance often included trial and error. Finally, participants described the centrality of *pushing back against diet culture and weight stigma* throughout the recovery process. Participants engaged in this pushback to various degrees. Diet culture and weight stigma had infused environments prior to and during the ED and recovery for most, and working into recovery was often scaffolded by a need to let go of weight as an arbiter of health.

### Theme 1: Expanding worlds: the process of exploring who I am

The concept of expansion was central to many participants’ stories. For both peer and family mentor participants, the world-in-recovery was described as bigger than what it had become during the ED. During the ED, many participants described a constrained, small, and isolated world where the ED was the main, or only, focus. The *expanded world* of recovery was often characterized by a more exploratory and joyous relationship with food and movement, as well as engagement with other interests and passions. In expanding recovery worlds, participants described building a new self; this new self could include aspects of the “person before,” but the ED, alongside other life and context changes, fundamentally impacted the self-in-recovery. Finally, expansion was not synonymous with “perfection” and recovery was not linear. Often, participants articulated a sense of self-compassion about living a life that aligned with their values, rather than one that entailed complete and total, final mastery over any and all negative thoughts.

As interests and activities expanded, participants’ relationship to those interests and activities often changed as well. Participant 303 (PM) illustrated how expansion was not always about big moments of shift, but rather could be noticed in micro-moments of change and identity development:

“It was refreshing. I think a lot of recovery for me was like identity, exploration, and like finding out who I am without the eating disorder so like finding… just like simple things out about myself like what's my favorite color, and like what *does* make me happy? And you know what are some things I'm interested in? Like, what are my favorite scents? Just silly small things, like… finding those things out about me just made me feel whole again. And it is… it is a fun experience just being like, wow, this is like this is fun finding out who I am.” (Participant 303)

Exploring these kinds of questions opened up ways of thinking about the self in a way that might be framed as “silly” or “small” but fundamentally related to the question of “who am I?” in recovery—a question others have suggested is core to disentangling the self from an ED [33]. For many peer mentor participants in particular, this question of “who am I?” (outside of the ED identity) led them to reflect on how recovery meant becoming someone new rather than going back to a pre-ED self*.* Participant 303 described this as feeling “whole again” (see also [31]); while they used the word “again,” which signals re-discovering the aspects of self that they had considered lost to the ED, they also described a *newness* to this experience of expansion and discovery. Both peer and family mentor participants reflected on how the ED and recovery process happened alongside other life changes, including normative developmental trajectories. Changes in self were attributable both to the experience of having and moving through an ED, as well as to broader life changes. To this sentiment, Participant 328 (PM) shared:

“It's funny [...] looking at my life, I feel like… just like a big shift. Like because that was what… like 6 years ago now? Like it feels like I'm a different person since then [...] I didn't necessarily feel like an old me, because that wasn't even what I was… that wasn't what my goal was. I had been in the eating disorder so long that I remember my mindset going into treatment was like, if there's a better version of my life that I need to, you know, try to experience that, or see if that's possible. So once I left treatment, I felt completely different. There was some interests that were, you know, still there in terms of like … things that I wanted to do and some of my professional goals. But I felt like a new sense of confidence, and a big motivation to actually pursue things that I wanted to do. [...] I felt like I had the room to actually think about it since I wasn't thinking about food all the time anymore.”

Participant 328’s quote illustrates how “returning to an old self” was not necessarily the goal of seeking treatment for their ED. Instead, they focused on treatment as an opportunity to see whether there was a way of being that would work better for them. This new way of being was not necessarily an *entirely* new self—some interests and goals remained the same. Many participants shared this orientation. For some, interests and goals had not changed during the ED, but the content or focus of those interests became largely about ED-related things. In recovery, the focus could once again be on things other than food and body. Thus, an expanded or different self was facilitated when food was no longer the central thing occupying one’s thoughts; this made space for exploration.

As noted, recovery itself was not necessarily the sole driver of the shifts and changes that accompanied this expansion and exploration. Participants were navigating recovery alongside growing up—often moving from being a teenager to being an adult. As Participant 302 (PM) shared:

“I feel a little bit, like and I've told this to patients having being in recovery as a young adult like… when your eating disorder starts as a teen, but now you're an adult it's not really getting back to a person it's, kind of… yeah… like building a new person like figuring out who you are. And so I think I never felt like I was back to my old self again. I just kind of realize, like oh, I'm a person. Now like, I have a personality and I have interests and friends.”

For many peer mentor participants, “building a new person” or becoming a new person involved trying new things and exploring—expanding. While a turning point featured in some stories, echoing the turning points described in other recovery literature [14, 43, 44], often a sense of steady change featured throughout these stories. Many of the changes participants described were related to broader life events and experiences, rather than necessarily related to food and exercise. This is not to say that relationships with food and exercise remained unchanged—many reflected on a significantly improved and genuinely enjoyable relationship with both. However, an exploration of change and expansion was often just as much, if not more, about non-food-related aspects of life: relationships, experiences, and identity.

Family mentors often reflected on the ways in which developmental and other life changes impacted the self they observed as their loved one moved into recovery. Perhaps due to their positioning relative to the person in recovery, however, they tended to remark on specific moments wherein they noticed shifts or the light or spark coming back. For example, Participant 322 (FM) noticed:

“Laughter, sense of humor coming back. She's a very funny person, and with a really witty sense of humor, and that was completely gone when she wasn't eating enough. So when she was able to respond to humor or make humor, that was really like the beginning of her coming back. There's another moment I remember. She was singing in the shower and her little brother could hear it, and he was like “[...] you have to come hear this.” Because it was like a really joyful thing, I know like singing in the shower can also be something like from residential where they make kids sing in the shower to make sure they're not like exercising or purging but that wasn't what this was. It was just spontaneous, like joyful singing and like even her little brother could sense that his sister was coming back.”

Many family mentors explicitly differentiated between their loved one and the ED itself, echoing an externalizing stance common in several ED treatment approaches, including narrative therapy [45, 46] and Family Based Treatment (FBT). Micro-moments like singing in the shower reflected glimmers of the person before the ED, reclaimed from the grips of the ED.

It is important to note that participants’ expanded worlds and new selves did not signal a kind of perfect recovery with no struggle at all. As life shifted and changed, so too would experiences in bodies and around food; for instance, a diagnosis of a medical condition might necessitate different relationships with food and body that challenged old ways of being. Further, many participants reflected on how being ok with things *not* being perfect was a sign of recovery. For instance, Participant 307 (PM) shared how:

“... recovery does not mean that you never dislike your body, or you never have urges, or you never want to do eating disorder things it’s just that you get to a point where you realize that's just not who you are, and it's not what you want to do, and for those reasons alone like you don't have to do it like you can just choose to live like with freedom and joy.”

Freedom and joy can be co-present with moments of disliking one’s body or having urges. The self-in-recovery, then, represented a self in the process of aligning with resonant values. Being in alignment with these values could help to anchor the joy and freedom this participant described. As we will discuss in the next theme, making choices related to “liv[ing] with freedom and joy” were not made in isolation, but often facilitated by having a supportive community that enabled this kind of flourishing (see also [15, 26]).

Many participants described how their “old self” was one that held some of the traits or characteristics that contributed to the development of their ED in the first place. For instance, participants described perfectionism, anxiety, people-pleasing, and high expectations for themselves. Participant 300 (PM) noted:

“Prior to the ED I had a lot of anxiety, was very anxious, was pretty introverted… had a lot of, I think social anxiety specifically and kind of, I guess. Like some like obsessive traits, as well as like, I think what's typically like type A personality. But kind of more to the extreme.”

Many described the “perfect storm” generated by these traits and environmental factors that impacted the development of their ED (see theme 3 for more on environmental factors). As such, many developed self-awareness and insight *about* these traits and how to relate to them in ways that would support recovery. The development of this self-awareness and insight was a part of the process of expansion that, for many, played a significant role in recovery experiences.

### Theme 2: Balancing autonomy and support in the recovery process

Giving and receiving support was a central theme throughout participants’ accounts of the recovery process. Core to this theme was the idea of finding a *balance* between support and autonomy; support is often central to recovery, but so too is the development of internal motivation [16]. Peer mentor participants described finding people who could support them as they developed a stronger ability to navigate their expanded worlds. Family mentor participants often described a sense of cautious optimism throughout the recovery process, and the material and emotional ways they worked to find supportive actions at different stages of the journey. Rebuilding broken trust—in others and in systems—was a core aspect of finding the balance between support and autonomy that enabled recovery.

Participants articulated how the ED often led to isolation, withdrawal, and secrecy. This sometimes led to trust with others being tenuous or broken. For family mentor participants, it was sometimes difficult to trust their loved ones again, as they were concerned that stepping back from their involvement in care might lead to precipitous ED relapse. Family mentor participants had often dropped everything to care for their loved one—or wished that they had been able to do so. Many attributed improvements in their loved one’s disorder to a combination of this wraparound support and the loved one’s engagement with the process. Because of the extent of their involvement in their loved one’s care, figuring out a way to move toward autonomy could be challenging and complex. As Participant 314 (FM) articulated:

“It's complex, because it's a traumatizing thing for a parent, for me to have gone through as well. So it's not like I felt… I see parents on the parenting boards that are like kind of gleeful or lighthearted and… great. [...] I think that for me it was a very cautious optimism… yeah, very cautious optimism, a gratitude. And, and… you know, still a commitment to vigilance.”

While family mentor participants were often grateful for the changes they saw in recovery, they sometimes continued to feel tentative about embracing the “glee” of this experience. Many described the experience as a whole as traumatic and difficult to let go of. As such, they noted that they remained committed to vigilance, or watchful engagement with the recovery process.

Several family mentor participants described how supporting their loved ones toward greater autonomy meant also recognizing that life would not be *perfect* or *flawless*. In this acknowledgement, they reflected on how a variety of challenges—related to or separate from food and eating—could arise. Participant 309 (FM) explained how:

"... and her life is, you know, like I respect her life, her 20 year old life. It's not a life that I would necessarily want going on in my home. So I think it's kind of a perfect in between. [Interviewer]: Yeah nice balance of like autonomy, and also like ensuring her wellness and welfare [Participant 309] Exactly.”

In describing “her 20 year old life,” Participant 309 reflected on how young adulthood can include a variety of challenges and experiences that they may not want to see first-hand. Instead, support could be delivered from more of a distance. Feeling comfortable enough to move to this greater level of autonomy was sometimes contingent on seeing the loved one engaging in self-reflection and insight, as Participant 313 (FM) described:

“It's like kind of an organic… I don't know, like an organic movement into this place of just trusting, knowing that she knew what she needed like to plate. And I think it's that plate by plate approach that like, taught her that you know what she needs for each meal and each snack throughout the day. And she just had a mindset of like knowing when she needed to fuel herself.”

This participant acknowledged the role that the treatment’s approach to eating (the plate-by-plate approach) played in teaching their loved one the skills to feed themself. In turn, when they saw that their daughter “had a mindset of knowing when she needed to fuel herself” they felt (organically) that their daughter was ready to move into a more autonomous stage of recovery.

Figuring out when and where to move toward autonomy from more wraparound support was a challenge many participants expressed. Several peer mentors noted that they wished they’d had a more gradual return to autonomy of eating, moving, and living, particularly in earlier treatment experiences after which they did not feel that they were in a solid place of recovery. This may be particularly important for those who develop EDs in childhood, which impacts what developing autonomy might look like [47]. For instance, Participant 308 (PM) noted that they had autonomy of eating “probably after that first year or less, I was in charge of my eating, which I would actually say is too early now looking back on it. But that's the reality.” Others had experienced a more gradual transition to autonomy of eating, which they described finding helpful, such as Participant 321 (PM):

“[...] it was kind of a gradual return to independence. So they kind of… I traded it down from like doing all of my meals and snacks to doing like some of my meals and snacks at treatment, and it's doing some at home. Sometimes I would like, bring in meals from home to treatment, so they could, you know, make sure I was doing everything I was supposed to, and then I started like not having that supervision and then transition to doing everything on my own when I discharged.”

The process Participant 321 described aligned with a more gradual transitional approach that many participants suggested was well suited to facilitating sustained change. Practical support, such as having support at the grocery store and learning to cook was helpful for some participants throughout the transition to autonomy. Having this kind of practical, hands-on support was not necessarily a common experience for participants, some of whom did not have significant support or treatment even during their eating disorder.

Several found engaging with supportive others who had similar lived experience to be a very helpful part of their journey. This form of support looked quite different from the more treatment-oriented, stepwise “transition from support to autonomy” characteristic of wraparound, step-down care. Instead, the relationship between support and autonomy played out over the course of the ED and recovery journey. Participant 304 (PM) described their more assembled approach to support and being supported:

“And I didn't really have like access to, like financial and health care, resources, or just any resources in that kind of vein to access, to identify, like needing treatment for myself. So much of the treatment recovery resources were centered around just kind of engaging in like free support groups and reading literature around eating sort of stuff.”

Participant 304’s experience of support might be described as more autonomous from the beginning, driven by engagement with free and accessible content and groups. They later described having “complete ownership of, you know, plating, and what I got to eat and chose to eat.” This autonomy could be helpful on the one hand, as it meant that the recovery process was self-motivated and driven. At the same time, they reflected on wishing that more helpful information had been readily available throughout their life; this, too, would have been a form of support.

Other peer mentors described how others with lived experience were instrumental in supporting their recovery in a way that enabled them to be themselves and feel seen and heard. Participant 326 (PM) noted that finding a community of people who “got it” was instrumental to recovery:

“[...] one of the things that really helped with recovery was I met other people who are also in recovery […] and so I kind of had like a community of people who got it, and I think that that made a big difference of people that I could support, and could support me as we like, move through recovery. But also, I think it helped reduce some of the shame because it's like, you know, “X, Y, and Z happened” like with behaviors, and it's like, “yeah, like I totally get it. It's hard to move through that”. And so there was much more understanding and compassion from others which helped me with having compassion with myself.”

Support, here, was configured as also shame-reducing; feeling understood and heard could be helpful in supporting self-compassion. Being supported, throughout participants’ accounts, could often help find ways of *supporting oneself*, in turn.

Finally, several participants shared how, as a mentor, being the person who could see and understand others felt rewarding and supportive of recovery. For instance, Participant 326 shared how

“It's part of why I'm so excited to work [in this role] … that's like right where I was. [...] I think it's so cool to be able to like… I don't know, be that space for people to talk in, or even just like being the person that has fun and enjoys things, and also is like recovering from an eating disorder” (Participant 326)

As Participant 326 further described, “being that space” for someone did not entail perfectly embodying a “recovered person,” but rather being someone who has been there, and was in the recovery process, but who also found joy in life. In turn, this helped them connect with their recovering self.

### Theme 3: Pushing back against diet culture and weight stigma.

This theme is focused on the role that weight stigma and diet culture played in ED and recovery experiences. Participants described experiences of shaming and/or fatphobic suggestions from medical providers that contributed to the “perfect storm” of an ED. Throughout the ED, participants described how they and their families needed to unlearn weight stigma and diet culture in order to support and sustain recovery. At times, more weight needed to be gained than initially suggested by medical providers. For peer mentor participants in particular, the need to let go of weight as a marker of health was often something that facilitated recovery. Some took this a step further, toward embracing a radical fat politic that sustained their being in the world.

Several participants described how a weight stigmatizing environment contributed to the onset of the ED. This was communicated in a nuanced way, wherein this stigmatizing sociocultural context was not *blamed* for the development of the ED; instead, it was one of several things that came together to generate a “perfect storm” of an ED (e.g., alongside personality and genetic factors). For Participant 326 (PM), this perfect storm also included food insecurity, illustrating the ways in which diet culture did not operate alone as an ED contributor:

“I definitely feel like a lot of my kind of eating disorder was almost a little bit of like a coping mechanism, like perfect storm situation between, you know, like 2000s diet culture and just kind of not having food around and trying to feel like I'm making the best out of it if that makes sense”

Here, Participant 326 described their ED as a “coping mechanism” as well as a perfect storm situation—a way to “make the best” of being food insecure and living in a society where diet culture ran rampant. Food insecurity is increasingly associated with eating disorders [48]; in this participant’s case, experiences of “not having food around” aligned with a diet culture mentality wherein food scarcity might be “made the best of.”

Other participants also commented on this time period (the early 2000s), reflecting on the prominence of pro-eating disorder and fatphobic, diet culture centric media and social media. For some, this contributed to a sense of being primarily valued for being thin. As Participant 304 (PM) put it, “creating a dynamic of […] if you're thin, or if you're like, at least striving to be thin…then you're good versus just like the whole valuing of people through their doing versus being.” The tying together of thinness and goodness participants described was, again, not a *cause* for their EDs, but rather a contributor both to the development of the eating disorder and an inhibitor to recovery as well.

Medical weight stigma could also be a part of this perfect storm; several participants described encounters wherein medical professionals prescribed particular ways of eating and moving that ended up being detrimental to health, positioning the person in a negative energy balance and further reinforcing societal weight stigma. Participant 322 (FM) described how:

“I wish at the 14 year check up my daughter's pediatrician hadn't said, you know, make sure you eat enough fruits and vegetables, and get enough exercise. Because she had gone like slightly above her growth curve. [...] I wish medical weight stigma hadn't been a part of this perfect storm”

Participant 322 reiterated that medical weight stigma was a part of the “perfect storm” their daughter had experienced that resulted in her ED. This part of the “perfect storm” added onto genetic and personality factors, as their daughter pursued a “healthy eating makeover” in her teens that led to a negative energy balance.

Weight stigma also led to diagnostic delays and/or non-diagnosis for several participants, who reflected on how misunderstandings of “the problem” led to mismatched treatment recommendations. Participant 327 (PM) shared:

“everybody just assumed that it was weight loss related that I need to focus on instead of actually getting treatment for an eating disorder. And so, even though I like would have had financial and like privileged access to treatment or like my parents would have absolutely like taken me to treatment, it was just never something even brought up or discussed, because the focus was always on my body size being smaller or needing to be smaller. So I think, in terms of like interventions or treatment options. It was like Weight Watchers and all of these different dieting options, and never eating disorder treatment so like on paper, I would have had access to it, but because of my body size it was something that I never had access to, because it wasn't even something I even knew about until I was […] in college, and realized what was going on.”

Participant 327 did not have their ED recognized until they were in college, long after onset. Like other participants, any attempts at weight loss were encouraged and even prescribed. Recovery involved not only unlearning but also teaching and educating others about the importance of not using body size as a proxy for health. For many, a high level of self-advocacy was required to avoid challenging situations around, for instance, being weighed or having problematic treatment suggestions made.

Many participants also reflected on how they felt that target weights had been set too low early on in treatment. In the large majority of these cases, participants described the provider or group who determined the target weight as lacking experience with ED’s. FM participants in particular tended to refer to how target weights were set too low, using normative data rather than individual growth curves. Having an inappropriate initial target weight set seemed to prolong the course of illness in many cases. For instance, Participant 312 (FM) said it would have been helpful “if we would have gotten to a higher weight quicker, maybe”; likewise, Participant 308 (PM) shared how

“you can't ignore the anti-fat bias in that [setting too low of a target weight range], and like why we're hesitant to even go a pound over the weight range. And I think, too, is to take the exhaustion I mean, we've fought for every… my parents fought for every pound that I gained in that time, and they were understandably like, oh, my God, we made it like we can stop now.”

People with EDs are often reassured that “they won’t get fat,” and target weight ranges are often set at a “low end.” At times, these practices impacted participants’ experiences of treatment, and limited the extent to which they could explore what it means to gain weight within a society that holds problematic views about fat bodies. Doing the work of letting go of weight as a meaningful marker of health happened outside of treatment settings for many participants. Participant 310 (PM) described feeling like letting go of the importance of weight felt more meaningful than the actual practice of weight restoration:

“in terms of like, like maintaining the weight wasn't as meaningful, and making me feel recovered, as like not doing certain rituals like I felt more recovered. Once I stopped, like, weighing myself or once I stopped compulsively exercising, that it made me feel more recovered than just the amount of time spent weight restored.”

This participant’s reflection questions the idea that length of time at a particular weight is an arbiter of recovery; instead, behaviors *associated* with weighing oneself felt like a more meaningful marker. This did not mean that weight restoration—and nutritional rehabilitation—were *not* important. However, this was often framed as a *starting point* versus an end point, particularly in a society where fatness, weight gain, and eating particular foods are vilified.

For some participants, developing a new appreciation of food beyond diet culture involved exploring cultural practices around food. Participant 308 (PM) reflected on how “I just have a stronger appreciation for food, being a really multifaceted thing. So food is also culture and food is also love and food is also fun. If I find a new recipe, I want to try, and it's not just numbers.” Food being associated with culture was not necessarily an easy experience for all; for Participant 301 (PM), the process of recovery also involved full family unlearning of particular cultural practices around food that did not support recovery. This participant shared how

“I think for my family, as a whole, to kind of unlearn some things that I think are common in just like [my culture …] like pushing food. And those kinds of things. It's just something we do. [...] there's still slips of things here and there but then being able to have like a conversation or calling in of like maybe let's talk about this was really helpful [...] it's been really helpful [...] it's still like a safe space to return to every time. I go home to my family to know that I don't have to really be concerned about like the diet culture really being as present as previously.”

Exploring the intersection of cultural practices around food and diet culture, Participant 301 reflected on the (what they described as scary) process of calling their family in to changing norms around food. Doing this work was not easy, but enabled a different kind of space where food could be eaten together in a way that did not play into diet culture while simultaneously honoring cultural ways of eating.

### Summary

In summary, we generated three themes from participant interviews aimed at better understanding the changes that occur in personality, traits, and interests over the course of an eating disorder and into recovery. In general, both individuals and caregivers observed changes in personality, traits, and interests during the acute eating disorder, which resolved or changed as they moved into recovery. Caregivers tended to describe more of a “return” to self, whereas those with an ED tended to describe a process of discovery of an expanded self and/or one that was more in line with their values.

## Discussion

Engaging deeply with lived experience perspectives from those who have first-hand experience of an ED and those who have supported loved ones with EDs helps to clarify the inner and outer “ingredients” of recovery processes. We set out to explore the shifts and changes that occur in personality, traits, and interests over the course of an ED and into recovery. In analyzing our data, we noticed that in exploring what it means to be “in recovery,” participants described a process of contraction in the ED and expansion into recovery. These processes were entangled with broader social contexts, including support and resources they did or did not have access to, and in relationship to diet culture and weight stigma.

These themes support a perspective that links individual experiences together with relational and systemic contexts in recovery. While the questions we asked invited introspection on personality, traits, and interests, participants’ stories extended far beyond an individual level analysis of their experiences. Recovery is entangled with social contexts, rather than being solely about individual functioning [13, 15, 26]. Finding supportive others—and particularly those who can help navigate cultural contexts rife with diet culture—can be instrumental in guiding processes of change [16]. Participants expressed the shifts in their journey as deeply linked to close others in their lives, as well as to their contexts—including experiences of food insecurity, cultural identity explorations, and weight stigmatizing contexts.

The “identity journey” of recovery often entails discovering new ways of coping and conceptualizing the self [14] and involves renegotiating who one is [5, 28]; participants expressed engaging in processes of discovery and exploration throughout their journeys as they engaged in this renegotiation. Discovering, or re-discovering, “who I am” has been noted to be a core component of recovery processes [33] as people embark on a journey of self-discovery [32]. For many participants, this self-discovery was tangled up in normative developmental trajectories, including negotiating autonomy and support. Finding this balance was not always easy or straightforward, and involved working through how the ED was one of many changes that impacted people and who they were becoming.

As participants in this study shared, constructing the self in recovery does not necessarily mean attaining a “perfect” way of being in the world. Rather than never struggling again, recovery may mean finding new ways of engaging with or interpreting this struggle. Being able to have compassion for oneself in this way was itself entangled with the process of expanding into recovery and discovering a sense of self that resonated with one’s values. The non-linearity and process-oriented view participants largely took on their recoveries has been shared throughout the literature [12, 16, 49]. Our findings join calls to conceptualize recovery, and the changes that occur throughout the process of recovery, as tied to social contexts and as more than a singular achievement or finished product [6, 13, 18, 19].

These findings also align with and extend literature that highlights how weight restoration is not an *adequate signifier* of recovery [8, 50]. The ways in which medical weight stigma and fatphobia can impede access to treatment, engender problematic treatment experiences, and hinder recovery efforts is increasingly acknowledged [13, 20, 51]. As such, our findings invite further investigation into how recovery definitions themselves may be constructed in ways that reinforce particular ideas about whose bodies are deemed recovered or “healthy.” That recovery represents swimming against a current of diet culture and normative discontent in bodies is likewise well-represented in the literature [17–19, 34]. In this study, we paid particular attention to how shifts in orientation to weight stigma, diet culture, and cultural norms around food and eating may occur across the course of an ED and into recovery—and how these shifts tied into the overall process of exploring the self in recovery.

Within the themes, we identified several nuances between peer and family mentor perspectives. While our methodology was not designed for direct comparison, we can explore how the perspectives brought forward by these differently situated participants add richness to the themes. For instance, peer mentors tended to emphasize a perspective on becoming someone *new,* rather than returning to an old self. Here, the ED had fundamentally changed them, and perhaps they did not even wish to return to the person before—the new person they built in recovery was more consistent with their values and current life stage. This resonates with a perspective that integrates illness-experience into identity in recovery in the process of reclaiming the self [14, 32]. While some vestiges of the old self remained, often the emphasis was on expansion into the new, described by many as an emergence of authenticity. Authenticity often meant living in line with one’s values, rather than an externally-defined idea about what a recovered self “should” look like.

Family mentors tended to focus more on how they noticed a *return*-to-self amongst their loved ones; they often noted specific moments of witnessing the spark or lights return to their child. While many noted that the self-in-recovery was a changed self, and that sometimes their loved one did not or could not return to prior interests, they continued to configure a core self that was something that could be returned to. This also has implications for the perceived vulnerability to relapse both groups described, but that family mentors tended to emphasize and that many feared. If a person is able to return to their old self, and this old self has personality traits that set the stage for the development of an ED, there is a higher degree of perceived vulnerability to EDs that remains. Participants in both groups did acknowledge their learning about potential vulnerabilities—and concordant need for self-insight and awareness to be a part of recovery. This seemed to particularly impact many family mentors’ orientations to the potential for the eating disorder to return—and perhaps led to increased vigilance and the kind of “cautious optimism” they described.

Weight stigma and diet culture can contribute to the development of EDs and also make recovery more challenging [13, 20]. Participants in this study often shared how they needed to unlearn diet culture and weight stigma—and how this was often something they did outside of the context of formalized treatment. Family mentors in particular nearly universally noted that treatment settings provided insufficient target weight ranges, though some peer mentors noted this as well. For peer mentors, there seemed to be a greater emphasis on the need to let go of the importance of weight in general—which was particularly challenging given the degree to which weight is employed in ED treatment contexts as a marker of illness and progress alike [13, 51].

Overall, having the perspectives of those with a personal experience and those with a carer perspective added richness to our findings. That we found similarities between perspectives shared by both sets of participants is in and of itself an important finding that is notable. This gestures at the potential for understandings of recovery shared between these groups but interpreted with attention to the added nuances that align with the unique vantage points participants brought to their stories.

### Scope of findings

Our findings must be interpreted in light of the specific participants we engaged with. Participants’ shared contexts and engagement with a single organization led to the development of an analysis about highly specific experiences of recovery. Peer and family mentors are in a unique position to be reflexive about their experiences; they engage with their stories regularly in order to support others seeking recovery. Further, mentors are “vetted” to ensure that they are in a place where they are able to provide support to others with EDs. In this process, there is a recognition of how recovery will not look the same for everyone, but equally an expectation that people providing support based on their lived experiences will have reached a place where they are doing well, as defined by the organization, in their own recoveries. In general, participants who take part in recovery studies may be especially keen to reflect on their experiences when compared to people in recovery who do not participate in studies, though this would be difficult to empirically confirm. The mentors interviewed in the current study may have been even more reflective around their experiences given the extent to which these experiences inform their day-to-day work. Further, the fact that all participants work at the same organization was evident in the language many participants used, which included terms like a “life worth living” and seeing the “lights come on.” However, this also aids in providing a richness and coherence to the data.

### Practice implications

There are several implications stemming from this work. Firstly, participants’ reflections on pushing back against weight stigma in order to support recovery invites practitioners to examine their own weight bias and stigma and how it impacts their practice. This may be particularly important when it comes to setting target weights. Many participants felt their or their loved ones target weights, often set by providers without ED specialization, were set too low, and often using normative versus individual growth data. This could impede or slow down recovery progress. Providers could benefit from additional training in non-weight-stigmatizing practice, as well as support in enacting build and ideological environments that are not weight stigmatizing. Providers might also be mindful about not setting target weights too low, and being sure to use individual growth curve data in setting weights vs. relying on population data.

Secondly, the complexity of support and autonomy articulated across participants’ accounts invites nuanced and honest conversations about the different types of support that may be helpful to people seeking recovery. It encourages a reconfiguration of the idea of recovery being an “in it for oneself” or individual process that calls on discourses of rugged individualism. In practice, this means thinking about the person-in-context and working collaboratively to ensure that standards for recovery set in treatment work in the context of the person’s life. This does not necessarily mean abandoning the metrics for recovery many use as touchpoints (e.g., symptom remission), but rather also taking seriously how aspects of life beyond symptoms may intersect with and impact someone’s life and recovery. Employing treatment approaches that work with the person to (re)discover who they are aligns both with the literature highlighting the importance of identity in recovery [14, 16, 33, 45] and our exploration of recovery processes.

Third, practitioners and researchers could consider recovery as ongoing and as more than an identifiable singular point in time (e.g., weight restoration) or finished product. Finally, the conceptualization of recovery as expansion invites an opening to the different ways in which recovery might expand in different directions—an openness to moving beyond singular ways of doing recovery. This might be achieved through truly collaborative care, including in determining what recovery means for the individual—and how the process of recovery might be best supported. This means moving beyond singular or “check box” orientations to recovery. Involving individuals with lived experience in the delivery of care can assist in realizing this goal, providing space for people with EDs and in recovery to be seen and heard in their full complexity.

### Supplementary Information

Supplementary Material 1.

## Data Availability

The datasets associated with this study are not publicly available as consent to publish full datasets was not sought and obtained from participants.

## Abbreviations

FBT: Family-based treatment
EDs: Eating disorders
BIPOC: Black Indigenous People of Color
