# Supplementary material for: “It’s like building a new person”: lived experience perspectives on eating disorder recovery processes
Source: J Eat Disord. 2024 Jul 8;12:96. doi: 10.1186/s40337-024-01045-5 (PMC11232212; doi:10.1186/s40337-024-01045-5)
Supplement: Supplementary file 1 — Supplementary Material 1. [file 40337_2024_1045_MOESM1_ESM.docx]

**Supplementary Materials - Interview Guide**

**Peer Mentor Interview Guide**

1. First, I’d like to know a little bit more about you and the language you use when it comes to your journey. This will help me know how to frame the remaining questions.

a. Specifically, can you share if there are parts of your identity or who you are that

feel especially relevant for me to know while we have this conversation?

b. What language do you use to describe your experiences with food and body?

Some people use “eating disorder”, “disordered eating”, or other language.

c. What language do you use to describe your experiences with healing? Some

people use “recovery”, “remission”, “recovered”, or other language.

2. Now, I’d like to learn about how you describe your eating disorder symptoms/history,

and treatment course (including levels of care, time in treatment).

d. Did you receive a diagnosis (or diagnoses)? If so, which one(s)?

e. How old were you when your eating disorder started?

f. What access to resources did you have during this time? Examples of resources

may be financial, housing, food security, education, social support, and affirming

care.

g. What age were you when you first entered recovery?

h. What access to resources did you have during this time? Examples of resources

may be financial, housing, food security, education, social support, and affirming

care.

3. How would you describe your personality or traits prior to the onset of the eating

disorder?

i. What kinds of personality traits did you notice in yourself?

j. How would other people have described you?

k. Does that resonate with how you describe yourself?

4. How would you describe your personality or traits while struggling with the eating

disorder?

l. Which traits and interests would you say stayed the same? Which traits and

interests changed?

m. How would other people describe ways you changed during this time?

5. What do you believe might have contributed to the changes you observed in your

personality during the eating disorder? In personality traits? In interests?

6. Was there a point that you felt like your “authentic self” or “old you” again?

a. What was that like?

b. How long did that take, if you consider the starting point as the onset of the eating

disorder?

c. What signaled to you that you were back to your ‘authentic self’/or ‘old you’?

Now I’d like to pivot to ask a few specific questions about that time around eating and

weight. Know that you don’t have to answer any questions you aren’t comfortable

answering.

d. What was eating like for you around the time you noticed the return of pre-eating

disorder traits? Please share as much or as little detail as you remember.

e. How would you describe the prescribed ‘meal plan’ at that time, if you had one?

If no specific meal plan was given, can you share any guidelines you recall being

given around eating at time of discharge?

f. How would you describe your weight at the time you noticed a return of your

‘authentic self/‘old you’?

i. How much did you weigh at that time?

ii. If a target weight/weight range was set as part of your tx plan, what was

the target weight/weight range?

iii. Were you ‘weight restored’? If so, for how long, and if not, how close

were you to being weight restored?

iv. Who determined/established your target weight/weight range, and what

was their area of specialization?

v. How tall were you at that time?

If you do not feel like there was a moment when you returned to your ‘authentic

self/old you’, how would you describe notable changes that happened in the

recovery process?

a. What was that like?

b. How long did that take?

c. What signaled to you that you were experiencing profound changes?

d. How much were you eating around the time you noticed the appearance of

post-eating disorder traits?

e. How would you describe the intake or ‘meal plan’ at that time?

f. How would you describe your weight at the time you noticed these changes? [Interviewer note: “We understand that you may not be aware of your weight history and related details, and you are welcome to share as little or as much as you'd be comfortable sharing"]

g. How much did you weigh at that time?

i. What was the target weight/weight range?

ii. Were you ‘weight restored’? If so, for how long, and if not, how

close were you to being weight restored?)

iii. Who determined/established your target weight/weight range,

and what was their area of specialization?

h. Could you please tell me a little about your physical activity engagement before, during, and after treatment?

i. Did physical activity impact the return of you to what you were like before the ED? If so, how?

7. How long did it take you to feel mentally healthy (from the onset of the eating

disorder)?

g. Could you please describe your status in treatment at that point

i. How long had you been engaged in treatment?

ii. Were meals supervised by caregivers at that point?

iii. How would you describe the patient’s level of autonomy around

meals/snacks, and how that transition to independent eating was made?

8. Did you struggle with lapses or relapses following a period of recovery? If so, how old

were you?

h. If so, can you describe any changes you observed in traits and interests?

i. What do you feel caused these changes?

9. Looking back, is there anything you would have done differently that may have allowed for an earlier return of your pre-ED personality traits?

10. Is there one thing (or several things) that you believe contributed most to the development of the eating disorder? If so, what is/what are these?

11. Is there one thing that you feel contributed most to recovery? If so, which one? Why?

12. How would you characterize your relationship with food and/or exercise now?

13. Please share anything else you feel is important for us to understand about your treatment/recovery journey.

**Family Mentor Interview Guide**

1. Could you please tell me a little bit about yourself.

a. Where do you live? How do you self-identify?

2. Next, I’d like to learn about how you describe your child’s eating disorder

symptoms/history, and treatment course (including levels of care, time in treatment).

i. Did your child receive a diagnosis (or diagnoses)? If so, which one(s)?

ii. How old was your child when their eating disorder started?

iii. What access to resources did you have during this time? Examples of

resources may be financial, housing, food security, education, social

support, and affirming care.

iv. How old was your child when they first entered recovery?

v. What access to resources did you have during this time? Examples of

resources may be financial, housing, food security, education, social

support, and affirming care.

3. How would you describe your child’s personality prior to the onset of the eating

disorder?

a. What kinds of personality traits did you notice in your child?

4. How would you describe your child’s personality/traits and interests while struggling

with the eating disorder?

a. Which traits and interests would you say stayed the same? Which traits and interests changed?

5. What do you believe might have contributed to the changes you observed in personality

during the eating disorder? In personality traits? In interests?

6. Now I’d like to pivot to talking about the time period when your child was in recovery. Was there a moment when you felt like your child was their authentic self again or back to their ‘old self’?

a. What was that like?

b. How long did that take?

c. What signaled to you that ’your child’ was back?

d. How much was your child eating around the time you noticed the return of pre- eating disorder traits?

e. How would you describe the intake or ‘meal plan’ at that time?

f. How would you describe your child’s weight at the time you noticed a return of your child?

i. How much did your child weigh at that time?

ii. What was the target weight/weight range?

iii. Was your child ‘weight restored’? If so, for how long, and if not, how close were you to being weight restored?)

iv. Who determined/established your target weight/weight range, and what was their area of specialization?

7. How long did it take your child to feel mentally healthy (from the onset of the eating

disorder)?

a. Could you please describe your child’s status in treatment at that point

i. How long had your child been engaged in treatment?

ii. Were meals supervised by caregivers at that point?

iii. How would you describe the patient’s level of autonomy around

meals/snacks, and how that transition to independent eating was made?

8. Could you please tell me a little about your child’s physical activity engagement before,

during, and after treatment?

a. Did physical activity impact the return of your child to what they were like before the ED? If so, how?

9. Did your child struggle with lapses or relapses following a period of recovery?

a. If so, what age(s) were they?

b. If so, can you describe any changes you observed in traits and interests?

c. What do you feel caused these changes?

10. Looking back, is there anything you wish would have gone differently that may have

allowed for an earlier return of their pre-ED personality traits?

11. Is there one thing (or several things) that do you believe contributed most to the

development of the eating disorder? If so, what is/what are these?

12. Is there one aspect(s) of treatment (or several) that you feel contributed most to recovery?

If so, which one(s)? Why?

13. How would you characterize your child’s relationship with food and/or exercise now?

14. Please share anything else you feel is important for us to understand about your child’s

treatment/recovery journey.
